# Supplementary material for: Prediction Models for Public Health Containment Measures on COVID-19 Using Artificial Intelligence and Machine Learning: A Systematic Review
Source: Int J Environ Res Public Health. 2021 Apr 23;18(9):4499. doi: 10.3390/ijerph18094499 (PMC8123005; doi:10.3390/ijerph18094499)
Supplement: Supplementary file 1 [file ijerph-18-04499-s001.zip › ijerph-1166403-supplementary.pdf]

Table S1. Search strings of databases

N	SCOPUS	SEARCH STRING	RESULTS
#1	SEARCH STRING FOR COVID	TITLE-ABS-KEY ((coronavir* OR (corona W/0 virus W/0 novel) OR HCoV OR nCoV OR covid* OR (corona W/0 infection*) OR (Severe PRE/0 Acute PRE/0 Respiratory PRE/0 Syndrome PRE/0 Coronavirus) OR ((SARS OR MERS) W/0 COV*) OR ((SARS OR MERS) W/0 Coronavir*) OR (Middle PRE/0 East PRE/0 Respiratory PRE/0 Syndrome) OR SARSS))	43,786
#2	SEARCH STRING FOR MODELS	TITLE-ABS-KEY (((theoretical OR mathematical OR stochastic OR deterministic* OR dynamic) W/0 (model* OR simulat*)) OR (intelligen* W/0 artificial) OR (learning W/0 machine))	2,148,857
#3	#1 AND # 2	TITLE-ABS-KEY ((coronavir* OR (corona W/0 virus W/0 novel) OR HCoV OR nCoV OR covid* OR (corona W/0 infection*) OR (Severe PRE/0 Acute PRE/0 Respiratory PRE/0 Syndrome PRE/0 Coronavirus) OR ((SARS OR MERS) W/0 COV*) OR ((SARS OR MERS) W/0 Coronavir*) OR (Middle PRE/0 East PRE/0 Respiratory PRE/0 Syndrome) OR SARSS)) AND ((theoretical OR mathematical OR sthocastic OR deterministic* OR dynamic) W/0 (model* OR simulat*))	845
#4	SEARCH STRING FOR PREVENTIVE MEASURES	TITLE-ABS-KEY ((Protective W/0 device*) OR (Face W/0 mask*) OR (Hand W/0 (hygien* OR wash*)) OR ((Respiratory OR Cough) W/0 (etiquette OR hygien*)) OR (Surfac* W/0 object* W/0 clean*) OR UV* OR (ventilation W/0 increas*) OR (humidi* W/0 Modif*) OR (Contact W/0 trac*) OR ((Social OR Patient* OR workplace OR school) W/0 (Isolat* OR Distanc*)) OR ((School* OR workplace* OR border) W/0 (measur* OR closur*)) OR lockdown OR (lock W/0 down) OR crowding OR ((Entry OR Travel*) W/0 (screen*)) OR (quaratin*) OR (outbreak W/0 (measure* OR containment OR Prevent*)))	791,743
#5	#1 AND # 2 AND # 3	TITLE-ABS-KEY ((coronavir* OR (corona W/0 virus W/0 novel) OR HCoV OR nCoV OR covid* OR (corona W/0 infection*) OR (Severe PRE/0 Acute PRE/0 Respiratory PRE/0 Syndrome PRE/0 Coronavirus) OR ((SARS OR MERS) W/0 COV*) OR ((SARS OR MERS) W/0 Coronavir*) OR (Middle PRE/0 East PRE/0 Respiratory PRE/0 Syndrome) OR SARSS)) AND (((theoretical OR mathematical OR stochastic OR deterministic* OR dynamic OR SEIR OR SIR) W/0 (model* OR simulat*)) OR (intelligen* W/0 artificial) OR (learning W/0 machine)) AND ((Protective W/0 device*) OR (Face W/0 mask*) OR (Hand W/0 (hygien* OR wash*)) OR ((Respiratory OR Cough) W/0 (etiquette OR hygien*)) OR (Surfac* W/0 object* W/0 clean*) OR UV* OR (ventilation W/0 increas*) OR (humidi* W/0 Modif*) OR (Contact W/0 trac*) OR ((Social OR Patient* OR workplace OR school) W/0 (Isolat* OR Distanc*)) OR ((School* OR workplace* OR border) W/0 (measur* OR closur*)) OR lockdown OR (lock W/0 down) OR crowding OR ((Entry OR Travel*) W/0 (screen*)) OR (quaratin*) OR (outbreak W/0 (measure* OR containment OR Prevent*)))	413

DATABASE	STRING	RESULTS
Nursing Reference plus	("coronavirus" OR "coronavirus infections" OR "coronavirus" OR "corona virus" OR "HCoV" OR "nCov" OR "covid" OR "covid19" OR "Severe Acute Respiratory Syndrome Coronavirus 2" OR "SARS-CoV2" OR "SARS-CoV 2" OR "SARS Coronavirus 2" OR "MERS-CoV")	53
Living evidence	((((intelligence) OR (machine)) OR ((model) OR (simulat)) AND ((theor) OR (math) OR (stoch) OR (determ) OR (dynamic) OR (SEIR) OR (SIR))) AND ((protective device) OR (Face) OR (Hand) OR (etiquette) OR (humidity) OR (air) OR (ventilation) OR (trac) OR (UV) OR (isolat) OR (distan) OR (school) OR (work) OR (border) OR (measur) OR (clos) OR (lock) OR (crowd) OR (travel) OR (screen) OR (quarant) OR (outbreak prevent) OR (outbreak containment) OR (outbreak prevent)))	1506
Pubmed	(((((("Models, Theoretical"[Mesh]) OR ("Models, Statistical"[Mesh])) OR ("Mathematics"[Mesh])) OR ("Mathematical Computing"[Mesh])) OR ("Artificial Intelligence"[Mesh])) OR (((((((((((model*[Title/Abstract]) AND (theoretical[Title/Abstract])) OR ("theoretical model*"[Title/Abstract])) OR (("theoretical model*"[Title/Abstract]) OR ((model*[Title/Abstract]) AND (theoretical[Title/Abstract])))) OR (((mathematical*[Title/Abstract]) AND (model*[Title/Abstract])) OR ("mathematical model*"[Title/Abstract]))) OR (stochast*[Title/Abstract])) OR (dynamic*[Title/Abstract])) OR (determ*[Title/Abstract])) OR (SEIR[Title/Abstract])) OR (SIR[Title/Abstract])) OR ("machine learn*"[Title/Abstract])) OR ("artificial intell*"[Title/Abstract])) AND ((((((((((("Hand Hygiene"[Mesh]) OR ("Hand hygiene"[Title/Abstract])) OR ((("Respiratory Hygiene"[Title/Abstract]) OR ("Respiratory etiquette"[Title/Abstract])) OR ("Cough etiquette"[Title/Abstract]))) OR (("surface* hygien*"[Title/Abstract]) OR ("surface* clean*"[Title/Abstract]))) OR (("Ultraviolet Rays"[Mesh]) OR (UV[Title/Abstract]))) OR (("Personal Protective Equipment"[Mesh]) OR (("facemask*"[Title/Abstract]) OR ("protective device*"[Title/Abstract])))) OR ((((("Social Distance"[Mesh]) OR ("Social Isolation"[Mesh])) OR ("Patient Isolation"[Mesh])) OR ("Quarantine"[Mesh])) OR ((((((("Social Distanc*"[Title/Abstract]) OR ("Social Isolation*"[Title/Abstract])) OR ("Patient Isolation"[Title/Abstract])) OR ("crowd*"[Title/Abstract])) OR ("Quarantin*"[Title/Abstract])) OR ("workplace"[Title/Abstract])) OR (school[Title/Abstract])))) OR ((((("Travel advice"[Title/Abstract]) OR ("travel screening"[Title/Abstract])) OR (("entry screening"[Title/Abstract]) OR ("exit screening"[Title/Abstract]))) OR (("travel restriction*"[Title/Abstract]) OR ("travel ban"[Title/Abstract]))) OR ((("border closure"[Title/Abstract]) OR ("lockdown"[Title/Abstract])) OR ("lock-down"[Title/Abstract])))) OR (("Contact Tracing"[Mesh]) OR ("contact trac*"[Title/Abstract]))) OR ("Increased Ventilation"[Title/Abstract])) OR (("policy making"[MeSH Terms]) OR (("outbreak"[Title/Abstract]) OR ("public health measure*"[Title/Abstract]))))) AND (("coronavirus"[MH] OR "coronavirus infections"[MH] OR "coronavirus"[TW] OR "corona virus"[TW] OR "HCoV"[TW] OR "nCov"[TW] OR "covid"[TW] OR "covid19"[TW] OR "Severe Acute Respiratory Syndrome Coronavirus 2"[TW] OR "SARS-CoV2"[TW] OR "SARS-CoV 2"[TW] OR "SARS Coronavirus 2"[TW] OR "MERS-CoV"[TW]) AND (2019/1/1:3000[PDAT]))	778

CINHAL	QUERY	RESULTS
S1	(MH "Coronavirus+")	1,078
S2	(MH "Coronavirus Infections+")	4,479
S3	coronavir* OR "corona virus" OR HCoV OR nCov OR covid* OR "Severe Acute Respiratory Syndrome Coronavirus 2" OR "SARS-CoV2" OR "SARS-CoV 2" OR "SARS Coronavirus 2" OR "MERS-CoV" OR "Middle East Respiratory Syndrome" OR SARSS	9,369
S4	S1 OR S2 OR S3	11,127
S5	(MH "Models, Theoretical+")	18097
S6	(MH "Artificial Intelligence+")	18,097
S7	S5 OR S6	197,941
S8	"models theoretical" OR "theoretical models" OR "mathematical model" OR "mathematical model" OR "Artificial Intelligence"	62,131
S9	S7 OR S8	201,068
S10	S4 AND S9	175
S11	(MH "Handwashing+")	9,418
S12	(MH "Protective Devices+")	2232
S13	(MH "Disease Outbreaks/PC")	6,497
S14	(MM "Quarantine")	244
S15	S11 OR S12	30,551
S16	S13 OR S15	36,746
S17	S14 OR S16	3692
S18	Facemask OR "Respiratory etiquette" OR "Respiratory Hygiene" OR "Cough etiquette" OR "Hand hygiene" OR "Surface objects Clean" OR UV OR "Increased Ventilation" OR "Modifying Humidity" OR "Contact tracing" OR "Social Distance" OR "Social Isolation" OR "Patient Isolation" OR "Isolation of sick individual*" OR "School measure*" OR "School Closure" OR "Workplace Isolation" OR "Workplace measure*" OR "Workplace closure" OR "Crowding" OR "Travel Advice" OR "Travel screening" OR "Entry scerrening" OR "Border Closure" OR "lockdown" OR "lock-down" OR "lock down"	31,064
S19	S17 OR S18	61,345
S20	S10 AND S19	38
